# Q-VAT: Quantitative Vascular Analysis Tool

**DOI:** 10.3389/fcvm.2023.1147462

**Published:** 2023-06-02

**Authors:** Bram Callewaert, Willy Gsell, Uwe Himmelreich, Elizabeth A. V. Jones

**Affiliations:** ^1^ Center for Molecular and Vascular Biology (CMVB), Department of Cardiovascular Sciences, KU Leuven, Leuven, Belgium; ^2^ Biomedical MRI Unit, Department of Imaging and Pathology, KU Leuven, Leuven, Belgium; ^3^ School for Cardiovascular Diseases (CARIM), Department of Cardiology, Maastricht University, Maastricht, Netherlands

**Keywords:** vasculature, quantification, density, morphometric analysis, immunohisthochemistry

## Abstract

As our imaging capability increase, so does our need for appropriate image quantification tools. Quantitative Vascular Analysis Tool (Q-VAT) is an open-source software, written for Fiji (ImageJ), that perform automated analysis and quantification on large two-dimensional images of whole tissue sections. Importantly, it allows separation of the vessel measurement based on diameter, allowing the macro- and microvasculature to be quantified separately. To enable analysis of entire tissue sections on regular laboratory computers, the vascular network of large samples is analyzed in a tile-wise manner, significantly reducing labor and bypassing several limitations related to manual quantification. Double or triple-stained slides can be analyzed, with a quantification of the percentage of vessels where the staining's overlap. To demonstrate the versatility, we applied Q-VAT to obtain morphological read-outs of the vasculature network in microscopy images of whole-mount immuno-stained sections of various mouse tissues.

## 1. Introduction

Structural alterations in the vasculature play an important role in several cardiovascular diseases (1–3). Studying the vasculature and its alterations is essential to get a better insight into the pathophysiological mechanisms during disease progression. There is a large variety of techniques to visualize and image the vasculature of different organs (4). Even though advanced imaging techniques are on the rise, classical histology is still considered the golden standard. High spatial resolution can be achieved which is highly beneficial for detailed imaging of complex structures such as the vascular network. However, there are several limitations in how classical histology is routinely applied in practice. Manual quantitative histological characterization of several samples is time consuming, which limits the amount of data that can be analyzed. Therefore, in practice often only a single or a few representative images are acquired and analyzed. This leads to sample bias which can result in incomplete or erroneous conclusions (5). Furthermore, it results in loss of most of the information from all the tissue that is stained but not imaged.

In recent years, technological improvements such as the development of whole-slide scanners have enabled the automatic acquisition of tiled images from large samples to full slides with a high spatial resolution. The acquisition is no longer constricted to certain manually selected regions of interest (ROI) (6). However, the large size of the obtained datasets creates new challenge with regard to archiving and data processing (7). Most image processing software's are designed to analyze complete images that can be fully loaded in the memory of the computer. Stitched high resolution images of large samples can produce images of several gigabytes that become too large to be loaded and processed directly. Since the microscopic images of large samples are captured as a series of smaller rectangular sub-images or tiles, these tiles can be extracted and used to perform the processing on each tile subsequently. This allows the processing of large datasets.

There are several tools available to automatically analyze and quantify the vasculature (8–15). Most of these existing tools rely on, often expensive, commercial software, require 3D images or are limited to a single image. While research institutes often have pay-per-use computers with such commercial licenses available, this is rarely applied to routine imaging of immunostaining. This limits the general accessibility and impedes their use for the automated analysis of tiled histological images. Furthermore, the morphometric read-outs are often extracted from the skeletonized vasculature, without considering the inherent limitations of skeletonization algorithms. They often introduce errors by normalizing per image rather than to the area of tissue. Lastly, though many phenotypes are limited to one vascular type (such as only in the capillaries), current quantification software rarely quantify with respect to vessel diameter.

We have developed Q-VAT (Quantitative Vascular Analysis Tool) to perform automated quantification of the vasculature in tiled, segmented two-dimensional images. Q-VAT is an easy to use tool written in the ImageJ macro language (16) that allows the user to automatically analyze and quantify the vascular network of large datasets in a tile-wise manner. When provided with a binary vascular mask and a tissue mask Q-VAT automatically calculates several morphological read-outs that characterize the vascular network. The vasculature can be divided into two compartments, allowing the user to focus on a certain compartment (e.g., the microvasculature), investigate whether a specific vascular compartment is affected in a certain pathology or exclude vessels above or below a certain threshold. Moreover, Q-VAT allows the addition of one or two co-staining's and automatically computes the ratios of co-staining compared to the first channel. When the analysis is finished Q-VAT automatically saves the quantitative morphological read-outs for each tile (into an Excel file) and creates tile-wise colormaps for each read-out. Q-VAT is freely available for download, together with a more detailed user guide on GitHub (https://github.com/bramcal/Q-VAT.git).

## 2. Materials and methods

### 2.1. Animals

Experiments were conducted in 102 weeks old (*N* = 4) B6CBAF1 hybrid mice obtained from Envigo. All experiments were approved by the KU Leuven Animal Ethics Committee. Experiments were performed according to the Belgian law (067/2008, 243/2013) and the guidelines from Directive on Care and Use of Experimental Animals (2010/63/EU) of the European Parliament.

### 2.2. Tissue processing & immunohistochemistry

Brain tissue was fixed in 4% paraformaldehyde for 24 h at 4°C and then stored in PBS with 0.1% NaN_3_. Next, the brain was embedded in 4% ultrapure low melting point agarose (16520050, Invitrogen) in PBS. The samples were cut into 40 µm coronal sections. Free-floating sections were stained with Lycopersicon Esculentum (Tomato) Lectin DyLight^TM^ 488 (1:1000, L32470, ThermoFisher). Mouse cardiac and liver tissue were fixed in 4% paraformaldehyde for 24 h at 4°C, paraffin embedded and cut into 4 µm (cardiac) or 7 µm (liver) sections. Cardiac sections were stained with Griffonia Simplicifolia Lectin I Isolectin B4 Dylight^TM^ 649 (1:100, DL-1208, Vector Laboratories). Liver sections were stained with primary Mouse endoglin CD105 (1:100, AF1320, R&D Systems) and secondary Cyanine 3 (Cy3 1:50, NEL704A001KT, Perkin Elmer) antibody. The eyes of the mouse were enucleated and fixed in 4% paraformaldehyde for 20 min at room temperature and then washed with PBS. The retina was dissected from the eye and further fixed in 4% paraformaldehyde for 24 h at 4°C. The free-floating retinas were stained with Griffonia Simplicifolia Lectin I Isolectin B4 Dylight^TM^ 649 (1:100, DL-1208, Vector Laboratories) and Collagen IV polyclonal antibody (1:200, 2150–1470, Bio-Rad) with secondary Rabbit IgG (1:400, A-31572, ThermoFisher).

### 2.3. Image acquisition

All samples were mounted on microscope slides and imaged using a Nikon NiE—Märzhäuser Slide Express two equipped with a Hamamatsu Orca Flash 4.0 camera. For each type of tissue, a custom-made JOBS-GA2 protocol was developed for sample detection. The brain sections were acquired as a *z*-stack with 5 planes at 5 µm separation with a Plan Apo 10× (NA 0.45) objective. The cardiac and liver sections were acquired as a single plane with a Plan Apo 20× (NA 0.75) objective. The retinas were acquired as a z-stack with 3 planes at 5 µm separation with a Plan Apo 10× (NA 0.45) objective. Before analysis, the Z-stacks were projected along the *Z*-axis using a maximum intensity projection and all files were converted to 8-bit and saved as Tag Image Files (.TIF). All input images used for this paper are available at https://doi.org/10.6084/m9.figshare.21820515.

### 2.4. Pre-processing

Excessive background signal and noise should first be reduced from the immuno-stained microscopy images. Depending on the quality of the staining and the type of tissue, masking of the background can be done using a simple thresholding or using more complex algorithms. The Q-VAT tool requires 8-bit binary TIF files with pixels belonging to the background set to 0 and pixels containing vasculature set to 255.

To ensure correct normalization of the quantitative morphological read-outs, a binary tissue mask is required (8-bit binary TIF image with background = 0 and tissue = 255). The tissue mask allows normalization to the tissue area, rather than the entire field of view of the tile. It also removes background signal originating from outside the tissue (e.g., fluorescent air bubble, dust particle) and allows empty tiles to be skipped during the analysis. Moreover, it allows the user to analyze only a specific ROI by providing defining the ROI through the tissue mask. In the GitHub page, we have included an ImageJ macro (Q-VAT masking tool) that uses a succession of several ImageJ commands to automatically create a vascular mask and tissue mask from stitched immuno-stained images (Supplementary Figure S1). The generation of these masks consist of two parts. First, the input image is used to create a tissue mask. Next, the input images and the tissue mask are used to create a vascular mask containing only the vasculature. Both masks are saved as whole images and as separate tiles that can be analyzed using the Q-VAT tool.

If the Q-VAT masking tool is used the input image is loaded and duplicated. One copy is used to create the tissue mask and the other to create the vascular mask. For the tissue mask, the contrast in the input image is first enhanced. Next, the image is smoothed to reduce noise, using a filter that replaces each pixel with the average of its 3 × 3 neighborhood. The resulting image is thresholded automatically to segment the tissue from the background using Huang's fuzzy thresholding method (17) and converted to an 8-bit binary image. Next, to fill remaining small holes, the image is median filtered by replacing each pixel with the median of the neighboring pixels using a user defined neighborhood radius (i.e., Radius for median filtering (µm)). Then, the “Analyze particles” command is used to obtain a binary tissue mask, where the small particles (e.g., ventricles) are removed based on a user defined threshold (i.e., particle size lower range (µm)). This results in an 8-bit binary image with pixels belonging to the background set to 0 and pixels containing tissue set to 255. This binary tissue mask is then divided into the original acquisition tiles. The tissue mask as a whole image and the separate tiles are each saved as TIF files. The user has the option to save a validation image, which consists of a superimposition of the vascular mask onto the original image. This feature facilitates visual inspection of the vascular mask.

The duplicated input image is used for the creation of a vascular mask. First, the input image is despeckled and smoothed. Next, the image is corrected for uneven background intensities using the convoluted background subtraction with a Gaussian kernel (BioVoxell Toolbox) (18). This background subtraction method creates a convoluted copy of the input image and subtracts the Gaussian filtered image from it. The radius for the Gaussian method (i.e., Radius of biggest object (µm) should be based on the radius of the biggest object in the input image. The background signal outside of the tissue is removed by multiplying the tissue mask with the background subtracted image. The resulting image is thresholded automatically to segment the vasculature from the background using automatic thresholding. The user is presented with a choice of thresholding methods, including ImageJ's default, Huang's fuzzy thresholding (17) and Otsu's thresholding (19).

### 2.5. Q-VAT pipeline

**Figure 1:**
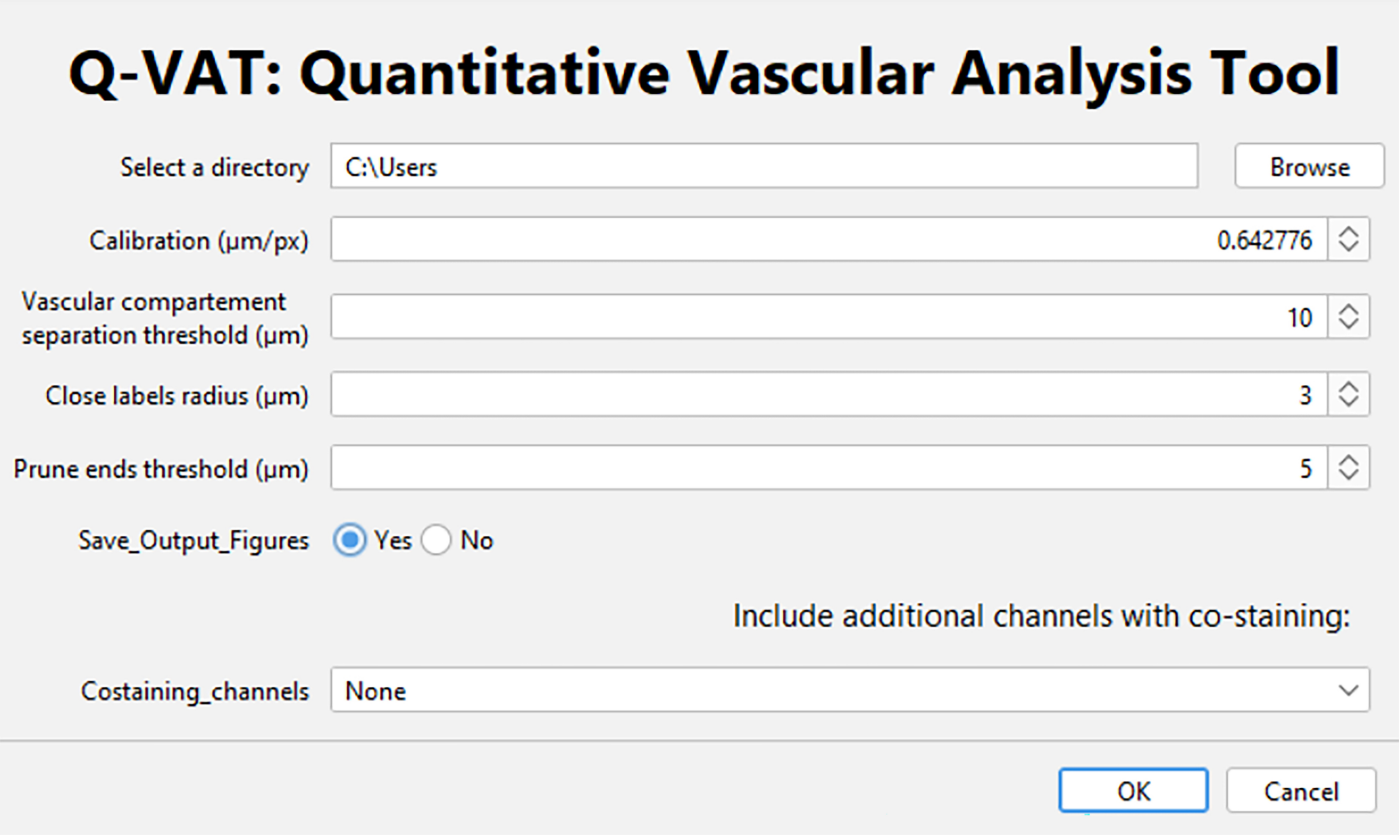
User interface of the Q-VAT tool. Through this interface the user selects the input directory containing the data to be analyzed, the spatial calibration (µm/pixel) as well as several input parameters: the vascular compartment separation threshold (µm), the close label radius (µm) and the prune ends threshold (µm). The user can choose whether or not to save the output figures. If multiple channels are to be analyzed, this can also be indicated.

When the pre-processing is performed using the Q-VAT masking tool, the files will be automatically saved according to the required file organization; The user only needs to provide the correct data directory and several input parameters in the user interface (see Figure 1). Q-VAT will automatically analyse and quantify all images contained within the designated data directory. More detailed instructions are available on the Github page (temporary link to be changed upon publication: https://github.com/bramcal/Q-VAT.git).

After the user input is complete, Q-VAT can be run on any operating system in which FIJI (i.e., ImageJ that has been bundled with several plugins) can be run to perform automated quantification of the vasculature in tiled, segmented two-dimensional images. The Q-VAT tool is entirely automated and requires no user intervention during the analysis.

Q-VAT loops over the different acquisition tiles and performs quantification of the vasculature based on the provided vascular mask and tissue mask. First, the tissue mask is loaded and converted to an 8-bit binary image. The number of pixels within the tile belonging to the background (Background = 0) and the tissue mask (Mask = 255) are determined. If a tile only contains background pixels, the image is closed and the next tile in the loop is loaded. If a tile contains pixels belonging to the tissue mask, the corresponding vascular mask is loaded and converted to an 8-bit binary image (Background = 0, Vasculature = 255). To remove background noise and only consider tissue within the provided tissue mask (or ROI), these two images are multiplied.

Intensity inhomogeneities, incomplete filling or inconsistent staining of the vasculature can lead to errors in the vascular mask, which will subsequently introduce errors in the quantification of the vasculature (e.g., incorrect vascular density, skeletonization errors). Therefore, an additional hole filling step is performed before the quantification. Small holes in the vascular mask with a diameter below a user defined threshold (i.e., Close label radius (µm)) are filled using the simple segmentation plugin followed by a closing operation (3D ImageJ Suite plugin) (20). The output images in the masked file subfolder can be verified for appropriate hole filling. The local thickness of the corrected vasculature is determined (Local Thickness) (21, 22) and the corrected vasculature is then skeletonized (Skeletonize (2D/3D)) (23). Small protrusions in the vasculature can result in incorrect endpoint branches, which will lead to quantification errors. Therefore, before analyzing the skeleton, endpoint branches with a length below a user defined threshold (i.e., Prune ends threshold (µm)) are removed from the skeleton (Prune Skeleton ends) (24). Both of these parameters (“Close label radius” and “Prune ends threshold “) are user defined (Supplementary Table S1). The diameter of the vasculature is determined by the software by measuring the local thickness along the center line of the vasculature. The diameter of the vasculature is encoded into the skeleton by multiplication of the local thickness by the skeleton. This results in a skeletonized image that contains the local thickness along the center line of the vasculature (i.e., the diameter) as intensity values. Because this generates a measure of the mean branch diameter, the vasculature can be divided into two compartments, based on a user defined threshold (i.e., Vascular compartment separation threshold). This allows the output to be separated by vascular compartment into vessels above and below a certain diameter (i.e., capillaries from larger vessels, or micro vs. macrovasculature).

This skeleton is then analyzed (Analyze Skeleton (2D/3D)) (24), which results in a range of vascular morphological read-outs: number of branches, number of junctions, number of endpoints, number of vessel clusters, branch length, average intensity (i.e., mean branch diameter), Euclidean distance. The branch length and the Euclidean distance are used to calculate the arc-chord ratio, that is used to estimate the tortuosity of the vasculature (Tortuosity Index = branch length/Euclidean distance). The mean diameter and the branch length are used to calculate the vessel area within the image (mean branch diameter*branch length = branch area). Normalizing the vessel area by the area of the tissue mask provides an estimate of the vascular density within the tissue (%). Several of the vascular morphological read-outs (mean vessel diameter, vascular density, vessel length density, mean branch length, branch density and tortuosity Index) are calculated for the branches above/below the user defined threshold, respectively. Definitions of all available measurements is provided in Supplementary Table S2.

Q-VAT allows the inclusion of up to two co-staining's (Supplementary Figure S2). For each of the channels, the percentage of the vascular area that is positive for the co-staining is calculated automatically. First, the non-overlapping signal are removed by multiplying the vascular mask of the first channel with the vascular mask of each of the additional channels. Next, Q-VAT will perform the same steps as described above on the overlapping signal from each of the additional channels. The ratio between co-stained channel and the vascular channel (first channel) will be calculated for each of the morphological read-outs. This functionality can be particularly useful for application such as empty collagen sleeves, the evaluation of the proportion of perfused vessels, co-localization of blood vessels with other structures (e.g., pericytes, vascular smooth muscle cells, nerve fibers) or the evaluation of different vascular staining methods.

### 2.6. Comparison of methods

For AngioTool, all images were imported, calibrated and analyzed with the default settings for vessel diameter (10) and vessel intensity (15–255). Foreground and background small particles were removed (483 px) and small holes were filled (12 px) with settings as close as possible to the input settings used for the Q-VAT analysis. Images containing the vessels outlines were exported using the overlay setting. Segmentation images were generated by filling the vasculature outlines in these images. Vascular morphological read-outs of the vascular density (i.e., vessels percentage area) and endpoint density (i.e., total number of end points/explant area) were obtained from the excel file containing analysis parameters and computed results. The branch density was calculated. The cluster density and branch density were calculated by counting the number of vessel clusters and branches in the segmentation and skeletonization images, respectively. These values were then normalized to the explant area, resulting in the cluster -and branch density (i.e., number of clusters/explant area; number of branches/explant area).

For REAVER, all images were imported, calibrated and analyzed in batch mode with the default settings for average filter size (128), wire dilation threshold (0), vessel thickness threshold (3). Segmentation of the vasculature was achieved using the grey to binary threshold (0.09) and small particles were removed similar to Q-VAT and AngioTool using the Minimum connected Component Area (483 px). The binary segmentation of all images was extracted. Vascular morphological read-outs of the vascular density (i.e., vessel area fraction), Branch density (i.e., segement count/field of view) were obtained. The endpoint density was calculated from the REAVER generated mat datafiles (i.e., endpoints/field of view). The cluster density was obtained by counting the number of vessel clusters in the binary segmentation images and normalizing to the field of view (i.e., number of clusters/fields of view).

We created an ImageJ macro to evaluate the segmentation generated by the different vascular quantification tools for the entire images. The performance of each quantification tool was evaluated by metrics that quantified the degree of agreement between the automatic segmentation and the manual segmentation, approximating the ground-truth. The segmentation's overall performance was assessed by calculating the Dice similarity coefficient, accuracy, sensitivity and specificity for the entire tile (9, 25) as follows:

$$\begin{array}{rl} & Dice\;similarity\;coefficient\;\;=\\ & \;\;\frac{2*true\;positive}{2*true\;positive+false\;positive+false\;negative} \end{array}$$

$$\begin{array}{rl} & Accuracy=\\ & \;\frac{true\;positive+true\;negative}{true\;positive+true\;negative+false\;positive+false\;negative} \end{array}$$

$$Sensitivity=\;\frac{true\;positive}{true\;positive+\;false\;negative}$$

$$Specificity=\;\frac{true\;negative}{true\;negative+false\;positive}$$

Where true/false positive/negative refer to the number of pixels in the entire image. Q-VAT yielded higher average Dice similarity, accuracy, sensitivity and specificity than the other two quantification tools (Supplementary Table S4).

### 2.7. Statistical analysis

Statistical analysis was carried out in GraphPad Prism 9. After quantification outliers were removed using robust regression and outlier removal (ROUT) method with a ROUT coefficient Q = 1%. All data values are given as mean ± SEM. Comparison between different ROIs were performed using one-way analysis of variance (ANOVA) with Tukey's test for multiple comparison. Group wise comparison between Q-VAT and existing automated vascular feature quantification tools was performed using one-way ANOVA with Holm-Šídák's test for multiple comparisons. Values of *p* < 0.05 were considered statistically significant.

## 3. Results

### 3.1. Method development

**Figure 2:**
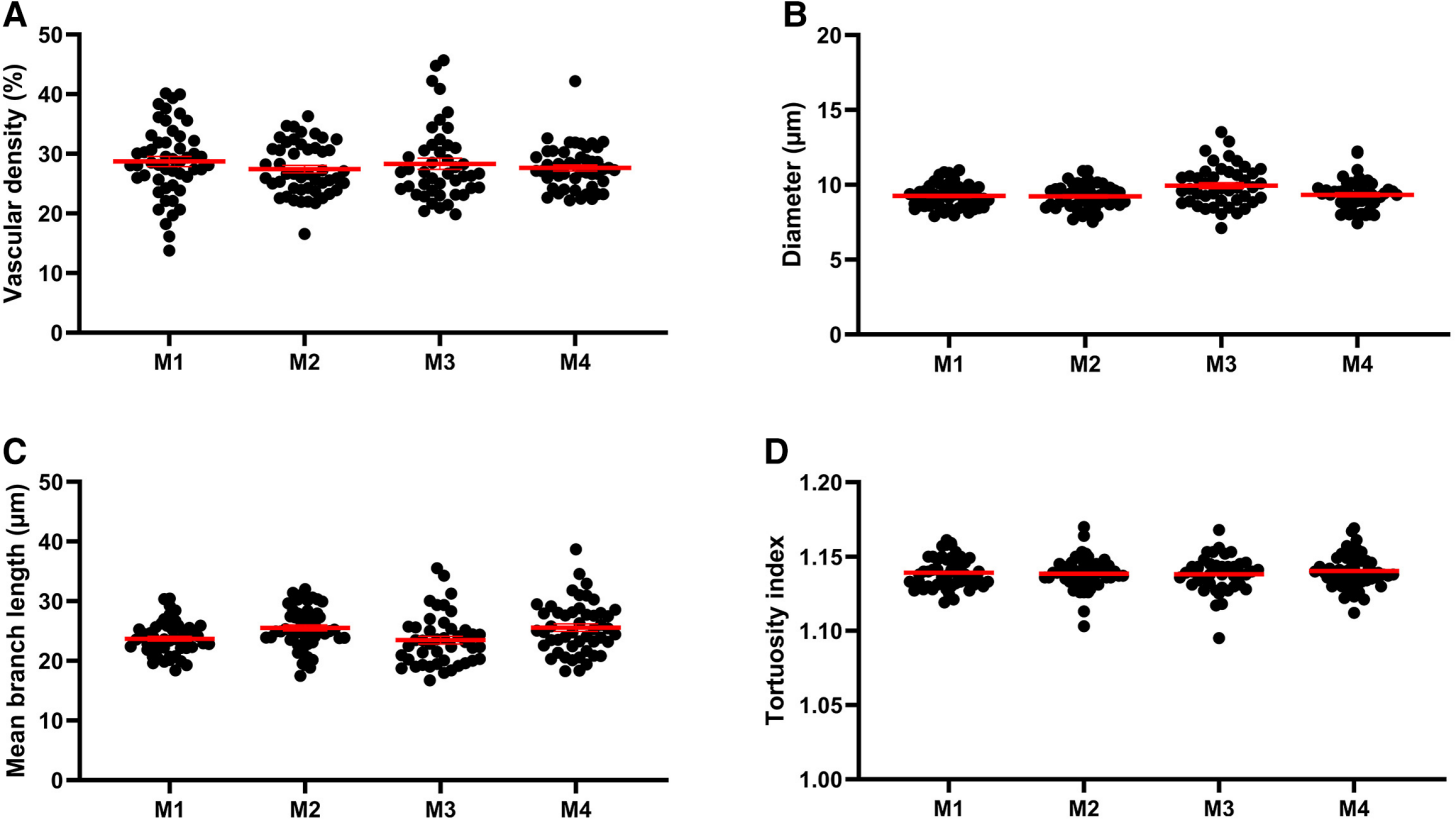
Morphological read-outs for 4 animals. Mean vascular density (average per tile) (**A**), mean vessel diameter (**B**), mean branch length (**C**) and tortuosity index (**D**) of the entire vasculature for each acquisition tile from the entire brain sections of each of the four mice (M1, M2, M3, M4).

Q-VAT was originally developed to perform automated quantification of the vasculature in entire two-dimensional brain sections. The stitched immuno-stained images of 40 µm thick brain sections were pre-processed to generate a vascular mask and a tissue mask. The following parameters were applied for the automatic analysis: Calibration, 0.643 µm/px; Vascular compartment separation threshold, 10 µm; Close label radius, 3 µm; Prune ends threshold, 5 µm. Figure 2 shows the mean vessel diameter, vascular density, mean branch length and tortuosity index of the entire vasculature for each acquisition tile from the entire brain sections of the four animals, which are averaged in Table 1.

**Table 1:** Average morphological read-outs for the entire brain sections of four animals (mean ± SEM).

Morphological read-out	All vessels	Diameter > 10 µm	Diameter < 10 µm
**Mean vessel diameter (µm)**	11.1 ± 0.3	13.5 ± 0.1	7.8 ± 0.1
**Vascular density (%)**	26.1. ± 0.3	17.1 ± 1.2	8.9 ± 1.1
**Vessel length density (mm/mm²)**	23.1 ± 0.8	12.2 ± 0.6	10.7 ± 1.4
**Mean branch length (µm)**	30.5 ± 0.6	28.9 ± 0.6	31.2 ± 1.6
**Branch density (#/mm²)**	766.1 ± 20.1	406.4 ± 11.7	341.0 ± 32.2
**Tortuosity index**	1.2 ± 0.0	1.1 ± 0.0	1.1 ± 0.0
**Cluster density (#/mm²)**	224.5 ± 14.5		
**Branchpoint density (#/mm²)**	282.4 ± 15.1		
**Endpoint density (#/mm²)**	665.1 ± 15.5		

### 3.2. Validation

**Figure 3:**
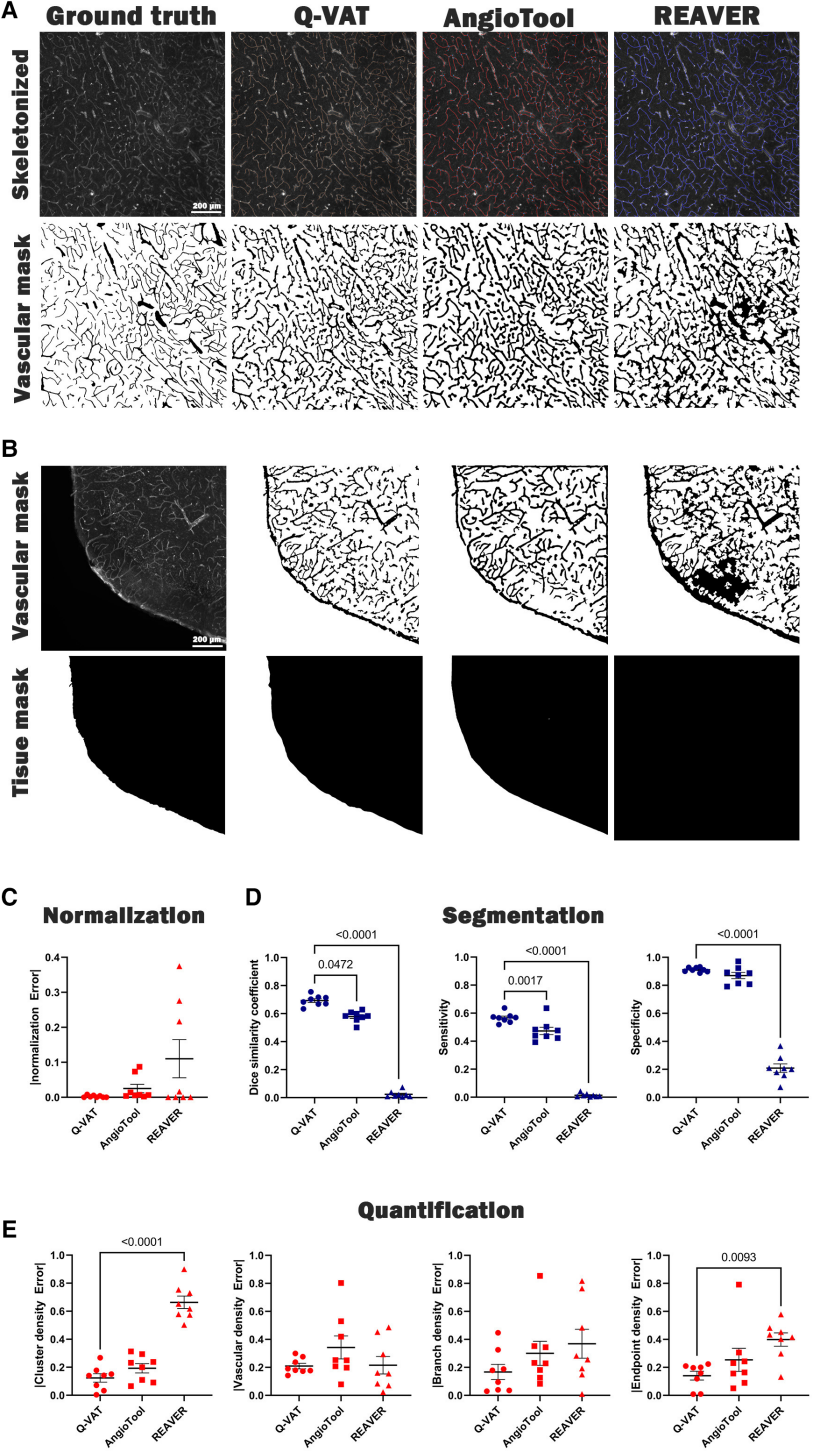
Validation of Q-VAT by comparison with existing methods for vascular feature quantification in microscopy images. (**A**) Representative image segmented **(bottom)** and quantified **(top)** using manual analysis, Q-VAT, AngioTool and REAVER. (**B**) Tissue masking of a representative images for all three methods. (**C**) Evaluation of the segmentation performance of the tissue area. (**D**) Evaluation of the segmentation performance of each quantification tool using Dice similarity (**left**), sensitivity (**middle**), and specificity (**right**). (**E**) Evaluation of absolute error comparing morphological read-outs (cluster density (#/mm²), vascular density (%), branch density (#/mm²) and endpoint density (#/mm²) to those obtained from manual segmentation.

We validated the measurements obtained with Q-VAT by comparison to existing methods for vascular feature quantification (Figure 3). Manual segmentation of the vasculature is widely considered as the gold standard method that yields most accurate segmentation. We therefore used manual segmentation as the benchmark, approximating the ground-truth, for evaluating the performance of automated segmentation. In order to validate the Q-VAT masking tool for pre-processing, the vasculature and the tissue area were manually segmented in eight randomly selected tiles, using ImageJ's paintbrush tool (Supplementary Figure S3). From the manual segmentation images, morphological read-outs of the cluster density (#/mm²), branch density (#/mm²) and endpoint density (#/mm²) were determined by manually counting the number of vessel cluster, vessel branches and endpoints. The vascular density was obtained by normalizing the number of pixels segmented as vasculature to the number of pixels manually segmented as tissue area. To evaluate the performance of Q-VAT for segmentation and quantification of the vascular network, we compared its output to those of two other tools designed to quantify the vascular network in fluorescent microscopy image, namely AngioTool (12) and Rapid Editable Analysis of Vessel Elements Routine (REAVER) (9) (Figure 3A). AngioTool is freely available open-source software written in the java programming language, designed to quantify the vasculature in microscopy images. REAVER is an open-source software tool written in MATLAB, designed to quantify the vasculature in high-resolution 2D fluorescent microscopy images. AngioTool was set to default conditions (see methods) and tiles loaded one-by-one since batch mode is not possible. REAVER was run in batch mode with a single grey to binary threshold per batch. REAVER allows manual correction of the segmented images, however this was not applied since it is not feasible for entire tissue sections.

Incorrect normalization of the morphological read-outs introduces errors in the quantification process. It is crucial to ensure that the normalization is performed only in relation to the tissue area. When analyzing images of larger samples, there are often areas of the image that are not completely covered by tissue. In such cases, accurate normalization becomes even more important to minimize the error introduced in the quantification process. The Q-VAT masking tool generates a tissue mask based on the intensity values of the original image, which is used to normalize the quantitative morphological read-outs (Figure 3B). AngioTool normalizes the read-outs to the area of the convex hull that contains all vessels in the input images. However, this approach can introduce normalization errors in cases where there are no particles at the edges or when the shape of the convex hull does not match with the actual shape of the edge of the tissue. REAVER, on the other hand, utilizes the entire field of view of the image to normalize the morphological read-outs. This can introduce normalization errors when analyzing images that are not completely covered by tissue. The normalization error was determined for each approach by calculating the absolute difference between the automated tissue segmentation and the benchmark segmentation of the tissue area (Figure 3B).

For our dataset of randomly selected tiles, Q-vat outperformed both AngioTool and REAVER in terms of segmentation performance for both the tissue area (Figure 3C) and the vasculature (Figure 3D). Dice similarity, accuracy, sensitivity and specificity (see methods for definitions) were highest for Q-VAT, however there was no statistical difference with respect to AngioTool except for Dice similarity and sensitivity (Figure 3D). AngioTool is much more labor intensive, however, requiring each tile to be uploaded and processed separately. REAVER was not developed for batches *per se*, and therefore fails to segment properly when the grey to binary threshold is not adjusted per image.

We assessed the performance of Q-VAT in analyzing and quantifying the vascular network by comparing the morphological read-outs from the different vascular quantification tools to those obtained from the manual segmentation (Figure 3E). The normalized absolute error with respect to the benchmark segmentation was calculated for the four morphological read-outs listed above. Q-VAT demonstrated lowest absolute error for all four morphological read-outs compared to the other two vascular quantification tools (Supplementary Table S5). These results demonstrate that Q-VAT is a reliable and accurate tool to quantify the vascular network in immuno-stained microscopy images of large samples.

### 3.3. Region-wise analysis

**Figure 4:**
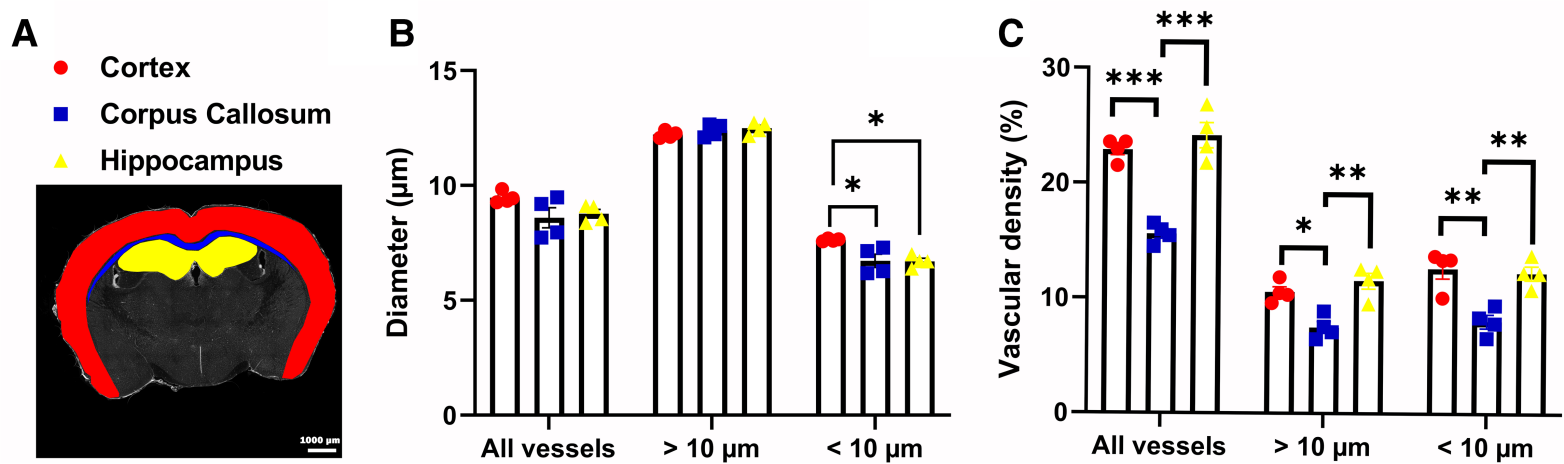
Region-wise analysis. (**A**) Manually delineated Regions Of Interest (ROIs) of the cortex (red), corpus callosum (blue) and the hippocampus (yellow) (*n* = 4). (**B**) Mean vessel diameter and (**C**) Average vascular density within the different ROIs for all vessels or vessels above/below a threshold (10** **µm). All data was analyzed with a one-way ANOVA and Tukey's HSD *post hoc* test. *p < 0.05, **p < 0.01, ***p < 0.0001.

Next, we demonstrated the ability of Q-VAT to perform region specific analysis. A mask containing a user-defined ROIs, rather than a tissue mask of the entire tissue section, can be used for quantification of specific regions of the tissue. The Q-VAT tool is limited to a single mask, but multiple ROIs can be analyzed by repeating the analysis using different input masks. We have manually delineated ROIs of the cortex (red), corpus callosum (blue) and hippocampus (yellow) in ImageJ and repeated the analysis for each ROI separately (Figure 4A). A significant reduction in the mean vessel diameter in the corpus callosum and the hippocampus was present as compared to the cortex (Figure 4B). This difference was not observed when considering all vessels, nor when considering only the vascular compartment above the separation threshold. The decrease in mean vessel diameter in the corpus callosum and hippocampus compared to the mean vessel diameter in the cortex can therefore be attributed to a reduction in the diameter of the smaller vessels in these ROIs. A significantly lower total vascular density was also present in the corpus callosum as compared to the cortex and hippocampus (Figure 4C), however this did not vary based on whether all vessels were analyzed or only a subset of all vessels. Similar differences in capillary density were previously reported in immunohistochemical staining's of mouse (26, 27) and rat brain sections (28, 29).

### 3.4. Different organs

**Figure 5:**
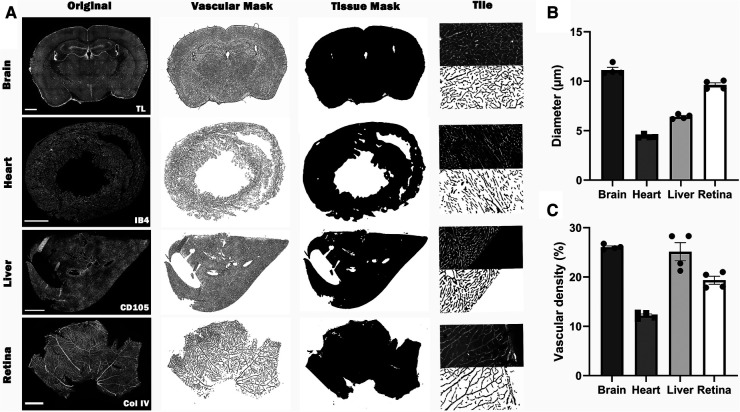
Overview of the automated quantification of the vascular network in immuno-stained microscopy images of different types of tissue using Q-VAT. (**A**) The original stitched high resolution images of the entire tissue section (first column, scalebars 1000** **µm) are pre-processed and used to create vascular masks (second column) and and tissue masks (third column). These masks are divided into the original acquisition tiles (last column; (top) half of an original acquisition tile; (bottom) segmented vascular mask of half of the acquisition tile) and are used to perform automated quantification of the vasculature. (**B-C**) Average morphological read-outs for the mean vessel diameter (**B**) and vascular density (**C**) (*n* = 4 animals per organ).

The application of Q-VAT is not limited to brain sections but can be used on sections from many different organs (Figure 5). For the retina, it was not necessary to split the image into tiles and the entire stitched image was analyzed as one tile. For the other organs, the obtained vascular mask and tissue mask were split up into the tiles matching the size of the acquisition tiles and analyzed in a tile-wise manner. The input parameters for the analysis of the presented data are reported in Supplementary Table S1, S3. The average vascular density and mean vessel diameter was higher in the brain and lowest in the heart (Figures 5B–C).

### 3.5. Tile-wise output maps

**Figure 6:**
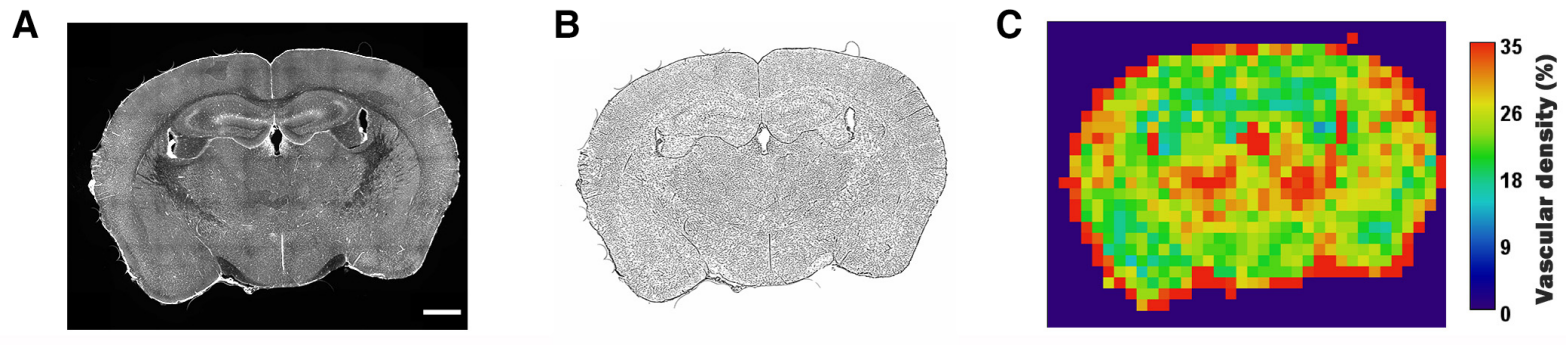
Example of a tile-wise morphological output map. (**A**) Original stitched high resolution images (scalerbar 1000** **µm), (**B**) Vascular mask, (**C**) Tile-wise output map of vascular density expressed in percent of tissue of the entire rat brain section. Area outside the brain tissue was set to pure black.

To create a visual readout of the tissue density, Q-VAT creates average tile-wise output maps for all measurements (Figure 6). In the previous sections, we have always performed the quantification on the original acquisition tiles. However, Q-VAT can also be performed on tiles with a different size. The tile-wise output maps offer a clear and simple way to visually represent the quantified morphological read-outs. Figure 6C shows an example of a tile-wise output map of the vascular density quantification of an entire rat brain section with a spatial resolution of 300 × 300 µm. There are several edge effects that should be considered when generating such tile-wise maps. One should be aware that partial volume effects will occur at the edges of the tissue (e.g., periphery and ventricles). Furthermore, non-specific staining at the edges of the tissue can occur due to peripheral drying, under fixation or buildup of secondary antibody (Figure 6A). These edge effects will introduce miscalculations at the edges of the sample (Figure 6C).

## 4. Discussion

We have developed a tool to perform automated quantification of the vasculature network in tiled, segmented 2D images in a free open-source software (Fiji). Furthermore, we provided an automated pre-processing pipeline that can be used to obtain the segmented vasculature from large immuno-stained microscopy images. Apart from the obvious advantages of automatic segmentation and quantification over manual quantification, Q-VAT addresses some of the limitations of other quantification tools. In contrast to other tools, Q-VAT was specifically developed to analyze tiled images. Thus Q-VAT can analyze large data files automatically, without the need to load each tile individually. This reduces the labor intensity of the vascular quantification. To reduce quantification errors, Q-VAT normalizes the morphological read-outs based on the area of tissue, rather than on the entire field of view. Furthermore, we addressed some of the inherent limitation of the skeletonization algorithms by filling small voids in the vascular images and pruning short endpoint branches in the skeleton. Additional morphological read-outs were added to the default skeleton analysis (Analyze Skeleton 2D/3D, ImageJ). A comparison of the absolute error between the results obtained with and without the additional correction steps was performed. Our findings revealed a statistically significant decrease in the absolute error when quantifying the branch density (#/mm²), while there was no significant difference observed in the other morphological read-outs (Supplementary Figure S4).

We validated the performance of Q-VAT along with two existing vascular quantification tools for both segmenting and quantifying the vasculature. To assess their performance, we compared the results to a benchmark data set of manually segmented images. Visual inspection shows that all automated quantification tools tended to produce thicker vessel segmentations compared to the ground truth. Therefore, when comparing values between different software tools, one should exercise caution and consider using relative vessel diameters to account for this effect. Using a dataset of randomly selected images, we demonstrated an overall improvement in segmentation performance compared to the existing vascular quantification tools. The average segmentation results provided by Q-VAT were in closest agreement to the manually segmented images, which are used as an approximation of the ground-truth. Furthermore, out of the three different automated vascular quantification tools, Q-VAT exhibited the smallest absolute error compared to the outputs obtained from the manually segmented images for all four morphological read-outs. The Q-VAT tool was specifically developed to automatically analyze immuno-stained microscopy images of large samples, which often exhibit a higher degree of background noise and intensity variability. The Q-VAT masking tool performs the pre-processing on the entire images rather than on individual tiles, which allows it to take advantage of the characteristics of the large samples. In contrast, the other quantifications tools were designed for analyzing individual images and may demonstrate better segmentation performance in scenarios where the image characteristics of the entire tissue sample cannot be leveraged. Furthermore, the images were analyzed using fixed input settings in AngioTool (individually) and REAVER (batch processing). Although adjusting the input parameters on an image per-image basis could potentially improve the segmentation slightly, this would be a time-consuming task when analyzing all tiles of large samples. The REAVER quantification tool provides the option to combine automatic analysis with manual curation. However, this approach is not feasible when analyzing the vasculature network of large tissue samples. Therefore, these options were not used in the method validation. The advantage of the Q-VAT tool is that it is entirely automated, allowing quantification of all tiles of a large sample without the need for manual intervention. This approach ensures the analysis of the entire tissue sample, avoiding information loss and sample bias. We can conclude that Q-VAT is a useful tool for the automated analysis of immune-stained microscopy images of large tissue samples.

A major advantage of Q-VAT over existing quantification software is its ability to divide the vasculature into two compartments based on the mean diameter of each branch and focus on a single vascular compartment. This allows Q-VAT to measure subtle difference that would otherwise have been undetectable. A limitation associated with this is that only a calculated estimate of the vascular density can be obtained. This means that Q-VAT does not measure vascular density directly but calculates it based on the diameter along the skeleton. As such, for vascular density, care should be taken when comparing values from Q-VAT directly with values measured by another software. However, it should be noted that despite this limitation, Q-VAT exhibited the smallest error in quantification of the vascular density in our dataset of randomly selected images.

Region-wise quantification of different brain regions demonstrated that Q-VAT is sensitive enough to detect changes in the morphological read-outs between different ROIs (Figure 4). Moreover, by dividing the vascular network into two compartments, based on the vascular compartment separation threshold, Q-VAT can observe significant differences in a specific vascular compartment, that would not be detectable if only the entire vasculature network is analyzed (Figure 4B).

Q-VAT reports all morphological read-outs for all tissues, however consideration should be given to which read-outs are suitable for specific types of tissue. Acquiring and analyzing only a single plane of a 3D structure, like the vascular network, will limit the accuracy of the quantification. The branching structure of the vascular network will not be visible and morphological read-outs can be biased by the slice thickness. Widefield microscopy allows imaging of relatively thin samples with a thickness within the depth of field. However, the depth of field typically decreases with increased magnification (due to larger apertures). This particularly becomes a problem when trying to image thick samples at a high magnification. To increase the thickness of the plane of focus and get closer to actual 3D acquisition, a series of images can be acquired along the axial direction (i.e., Z-stack). Combining these images into a 2D image using a projection removes out of focus information and provides a more realistic 2D representation of a 3D structure.

Since Q-VAT splits the stitched high-resolution images into smaller tiles, the large blood vessels will also be divided into pieces. Due to this inherent limitation, the length quantification of long blood vessels running across different tiles will not be correct. This will also be the case when quantifying only a few manually acquired representative images. The smaller the analysis tiles, the larger the effects on the length quantification will be. However, the same error occurs when quantifying only a few manually acquired representative images. This effect will be reduced when focusing only on the microvasculature (using the vascular separation threshold), as the mean branch length of the microvasculature is much shorter compared to larger vessels.

Q-VAT is limited to 2D images, or 2D projections of Z-stack images. Others have developed software tools for 3D vascular beds (30–32). Considering most research groups working with mouse or human tissue samples are still performing 2D imaging on sections, we focused our efforts on splitting the analysis based on vessel diameter. Expanding this tool to image quantification of 3D vascular networks would be the logic next step in the further development of Q-VAT.

## Data Availability

The datasets presented in this study can be found in online repositories. The names of the repositories and accession number(s) can be found in the article/**Supplementary Material**.
