# Supplementary material for: Q-VAT: Quantitative Vascular Analysis Tool
Source: Front Cardiovasc Med. 2023 Jun 2;10:1147462. doi: 10.3389/fcvm.2023.1147462 (PMC10272742; doi:10.3389/fcvm.2023.1147462)
Supplement: Supplementary file 1 [file Datasheet1.pdf]

Supplementary Material

1 Supplementary Data

The datasets used for this study can be found online at: https://doi.org/10.6084/m9.figshare.21820515

Supplementary Data 1-4. Whole mount coronal mouse brain sections (M1-M4) used for analysis shown in Figure 2-5.

Supplementary Data 5-8. Whole mount cardiac sections (M1-M4) used for analysis shown in Figure 5.

Supplementary Data 9-12. Whole mount liver sections (M1-M4) used for analysis shown in Figure 5.

Supplementary Data 13-16. Whole mount Retinal sections (M1-M4) used for analysis shown in Figure 5.

2 Online repository

Source code for the Q-VAT (Fiji) ImageJ and is available for download, together with more a more detailed user guide on https://github.com/bramcal/Q-VAT.git.

3 Supplementary Figures

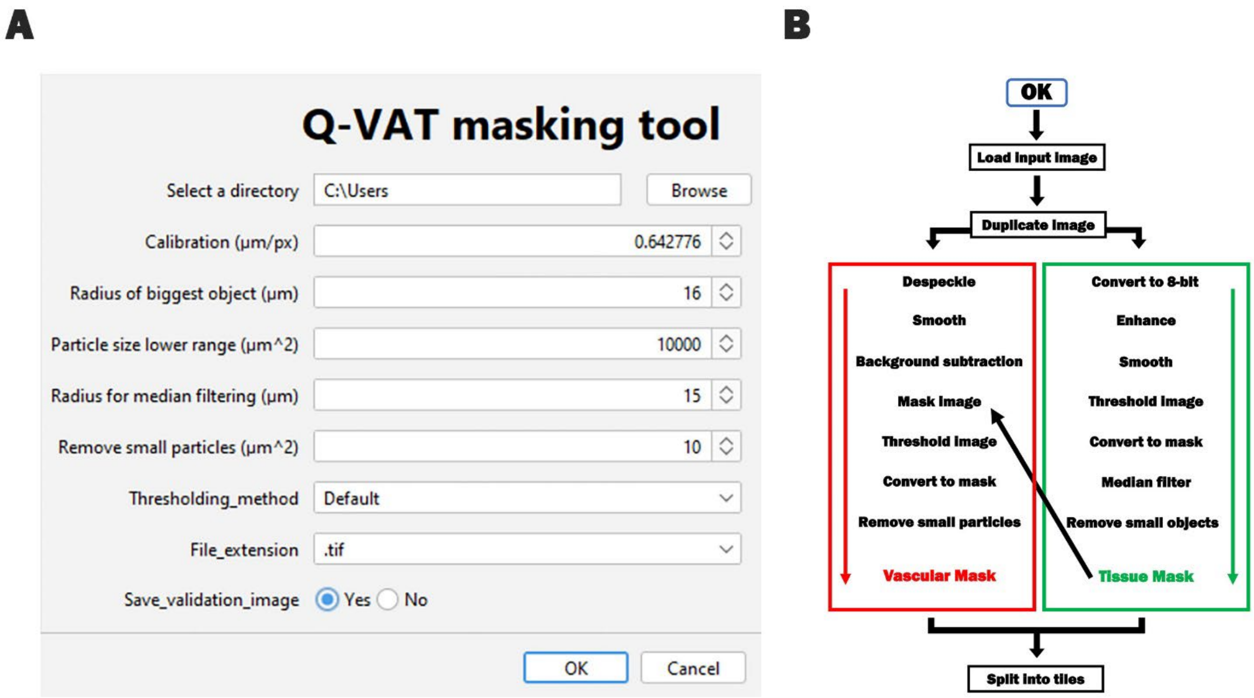

Supplementary Figure 1. Overview of the Q-VAT masking tool for pre-processing. **(A)** User interface and input parameters of the Q-VAT masking tool. Through this interface the user selects the input directory containing the data to be pre-processed, the spatial calibration (µm/pixel) and various input parameters: the radius of the biggest object (µm), tissue mask particle size lower range (µm²), radius for median filtering (µm), area of the small particles that should be removed from the vascular mask (µm²) and the thresholding method (Default, Huang or Otsu). The user can choose whether or not to save a validation image. **(B)** Schematic diagram showing the ImageJ commands performed by the Q-VAT masking tool to automatically create a vascular mask and tissue mask.

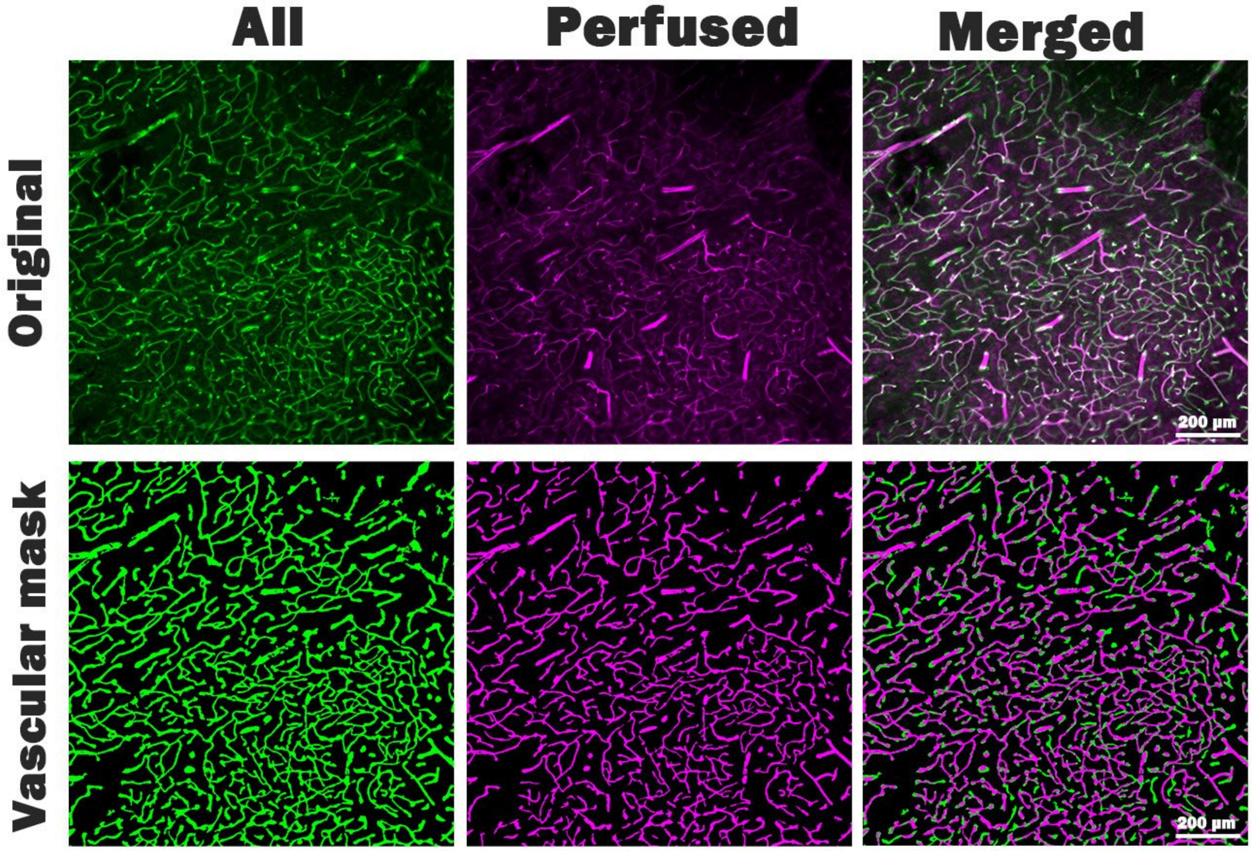

Supplementary Figure 2. Example of the co-staining functionality, which allows the user to add one or two co-stainings and calculate co-staining ratios. Original fluorescence microscopy of all vessels in the tissue (1:1000, L32470), the perfused vessels using an injection of Biotinylated Lycopersicon esculentum Lectin (B-1175) and the merged image **(Top)**. Vascular mask obtained using the Q-VAT masking tool for each channel **(Bottom)**.

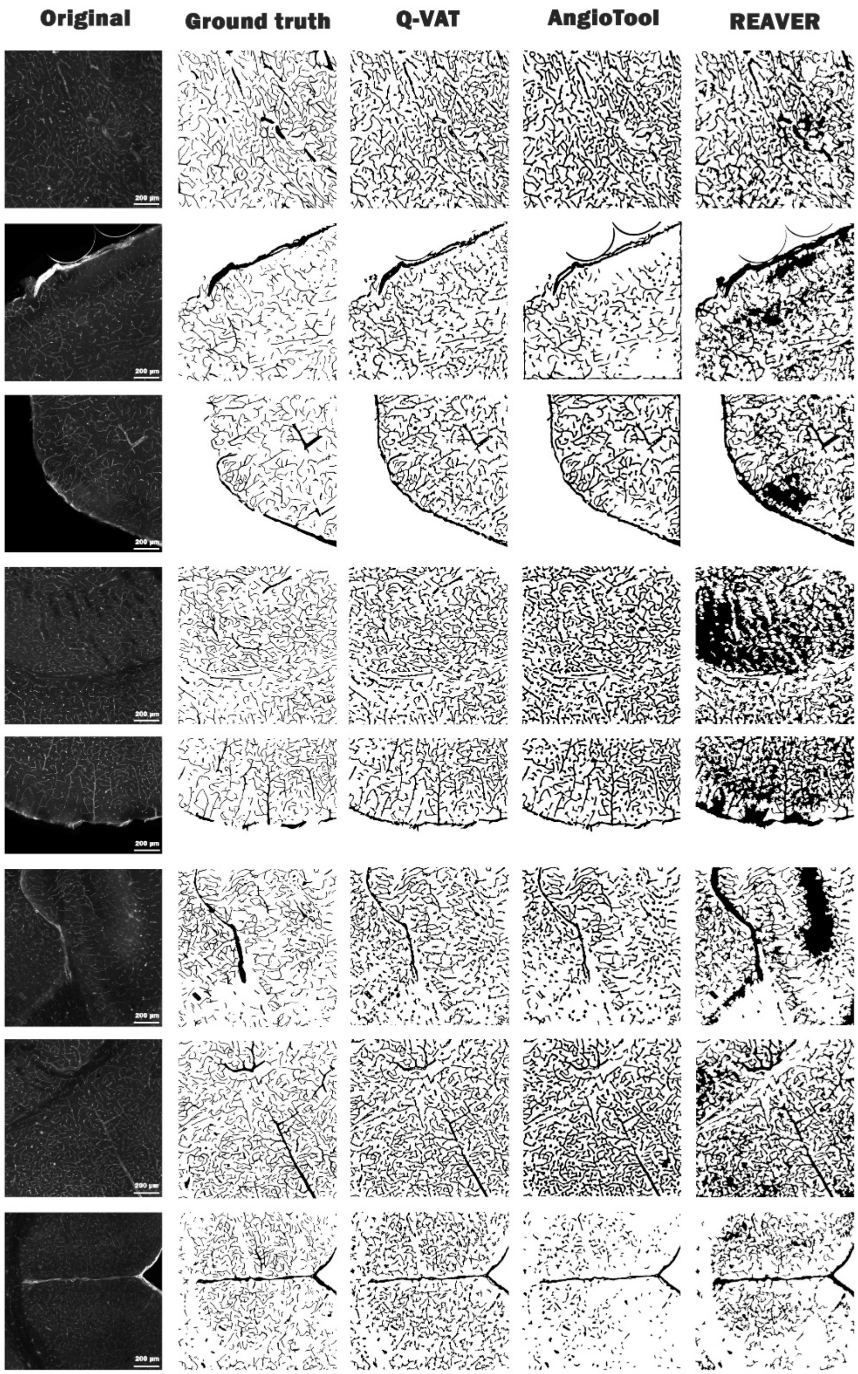

Supplementary Figure 3. Dataset of randomly selected tiles used for evaluating the performance of the automated segmentation. The first column shows the original fluorescence images, while the second column displays the manual segmentation results used as an approximation of the ground truth. The manual segmentation images were compared to the automated segmentation obtained using the different methods for vascular feature quantification. Automated segmentation results using Q-VAT, AngioTool, and REAVER are shown in the third, fourth, and fifth columns, respectively.

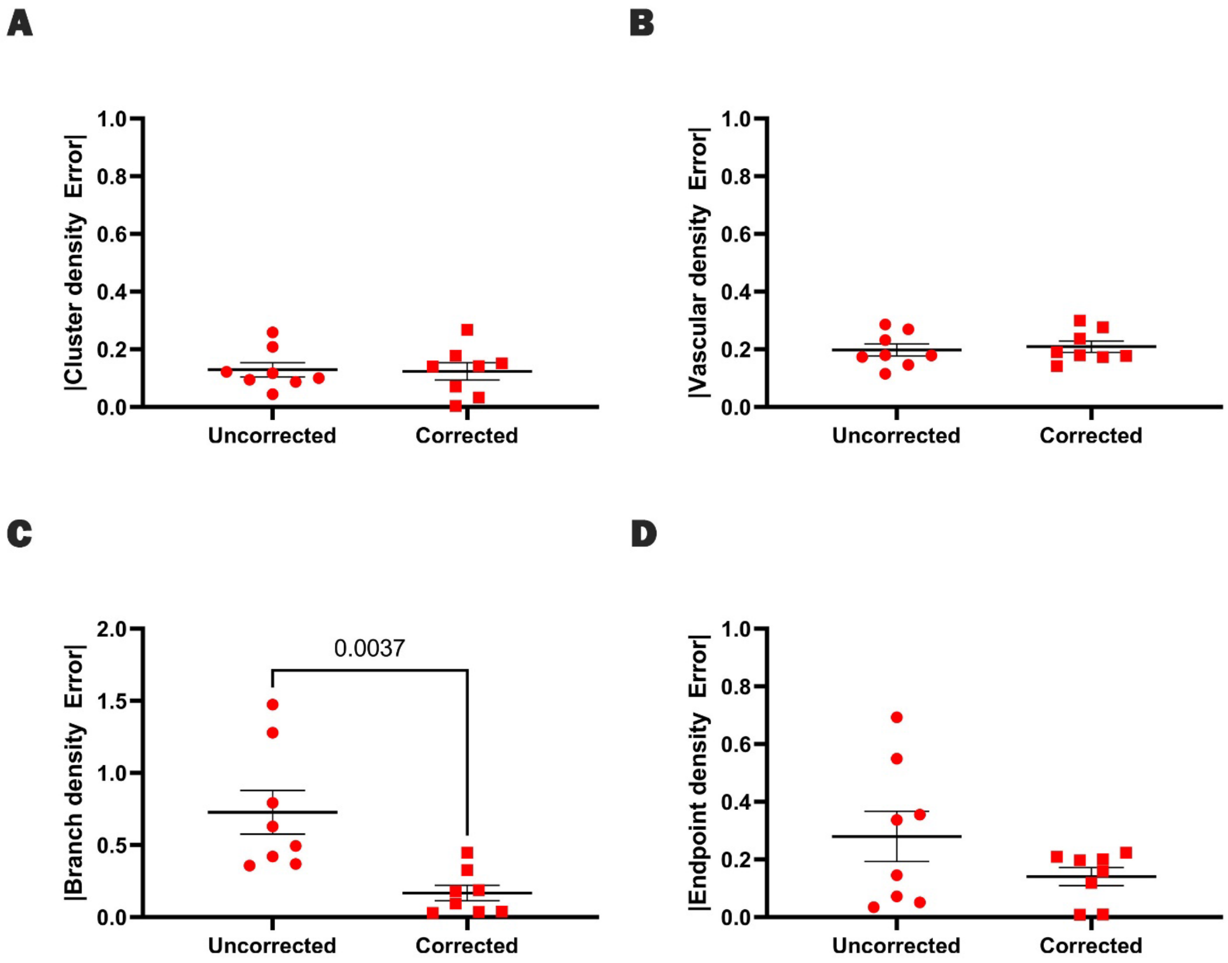

Supplementary Figure 4. Comparison of the morphological read-outs without (uncorrected) and with (corrected) the addition of small hole filling and pruning of small protrusions in the vasculature for **(A)** cluster density (#/mm²), **(B)** vascular density (%), **(C)** branch density (#/mm²) and **(D)** endpoint density (#/mm²). Values are presented as mean absolute error ± SEM. Two-way tailed t-test were used for group-wise comparisons, and statistical significance was determined at $p<0.05$. were considered statistically significant.

4 Supplementary Tables

Supplementary Table 1. Input parameters Q-VAT masking tool.

Input Parameter	Values			
	**Brain**	**Heart**	**Liver**	**Retina**
**Pixel calibration (µm/px)**	0.643	0.322	0.322	0.643
**Radius of the biggest object (µm)**	16	16	16	16
**Particle size lower range (µm²)**	10000	10000	10000	10000
**Radius for median filtering (µm)**	15	15	15	15
**Remove small particles (µm²)**	200	10	10	50
**File extension**	.tif	.tif	.tif	.tif

Supplementary Table 2. Overview of the Morphological read-outs computed by Q-VAT and their description.

Morphological read-out	Description
**Mean vessel diameter (µm)**	Average of the mean vessel diameter of all branches in the tile
**Vascular density (%)**	Area within the tissue that is covered by vasculature
**Vessel length density (mm/mm²)**	Total vessel length within the tile normalized by the tissue area
**Mean branch length (µm)**	Average of the mean branch length within the tile
**Branch density (#/mm²)**	Number of branches normalized by the tissue area
**Tortuosity index**	Average arc-chord ratio (i.e. ratio between the branch length and the Euclidian distance)
**Cluster density (#/mm²)**	Number of vessel clusters normalized by the tissue area
**Branchpoint density (#/mm²)**	Number of branchpoints normalized by the tissue area
**Endpoint density (#/mm²)**	Number of endpoints normalized by the tissue area

Supplementary Table 3. Input parameters Q-VAT.

Parameter	Values			
	**Brain**	**Heart**	**Liver**	**Retina**
**Pixel calibration (µm/px)**	0.643	0.322	0.322	0.643
**Vascular compartment separation threshold (µm)**	10	10	10	10
**Close label radius (µm):**	3	3	3	3
**Prune ends threshold (µm)**	5	5	5	5

Supplementary Table 4. Evaluation of automated segmentation performance for the different vascular feature quantification methods, based on accuracy, sensitivity, specificity, and absolute error of tissue area.

Quantification methods	Performance metrics				
	**Dice similarity coefficient**	**Accuracy**	**Sensitivity**	**Specificity**	**|Normalization Error|**
**Q-VAT**	0.693 ± 0.013	0.910 ± 0.005	0.566 ± 0.012	0.911 ± 0.006	0.002 ± 0.001
**AngioTool**	0.579 ± 0.014	0.865 ± 0.014	0.473 ± 0.026	0.870 ± 0.022	0.025 ± 0.012
**REAVER**	0.023 ± 0.0008	0.195 ± 0.024	0.014 ± 0.005	0.209 ± 0.031	0.110 ± 0.054

Supplementary Table 5. Performance evaluation of quantification of the vascular network for four morphological read-outs for the different vascular feature quantification methods using the normalized absolute error wit respect to the manual benchmark segmentation.

**Quantification methods**	**Absolute error**			
	**|Cluster density Error|**	**|Vascular density Error|**	**|Branch density Error|**	**|Endpoint density Error|**
**Q-VAT**	0.123 ± 0.030	0.209 ± 0.020	0.167 ± 0.054	0.141 ± 0.031
**AngioTool**	0.193 ± 0.033	0.342 ± 0.082	0.300 ± 0.086	0.254 ± 0.082
**REAVER**	0.663 ± 0.045	0.215 ± 0.062	0.369 ± 0.103	0.399 ± 0.047
